# Flexible NiCr–NiSi Thin-Film Thermocouple Sensor for Temperature Monitoring of Telecommunication Equipment

**DOI:** 10.3390/mi17060735

**Published:** 2026-06-18

**Authors:** Ruihan Gao, Jiaen Zhou

**Affiliations:** 1 International School, Beijing University of Posts and Telecommunications, Beijing 100876, China; 2 School of Information and Communication Engineering, Beijing University of Posts and Telecommunications, Beijing 100876, China

**Keywords:** flexible thin-film thermocouple, NiCr–NiSi, temperature monitoring, telecommunication equipment, dynamic thermal measurement, rapid response

## Abstract

Reliable temperature monitoring is essential for the thermal management and safe operation of modern telecommunication equipment. However, conventional temperature sensors are often relatively large and rigid, which limits their applicability for localized temperature measurement on compact electronic components. In this study, a flexible thin-film thermocouple based on NiCr–NiSi thermoelectric materials was developed for temperature monitoring of telecommunication equipment. The sensor adopts a multilayer structure consisting of a polyimide (PI) flexible substrate, an Al_2_O_3_ insulating layer, NiCr and NiSi thermoelectric films, and a SiO protective layer and was fabricated using magnetron sputtering. Static calibration experiments show that the fabricated sensor exhibits a thermoelectric sensitivity of approximately 40.45 µV/°C, which is close to the reference value of conventional K-type thermocouples, with a relative error of about 1.34%. Repeated heating–cooling cycles demonstrate good repeatability and stable thermoelectric characteristics. Dynamic tests under representative transient thermal conditions showed that the sensor could continuously capture temperature variations without signal interruption or abnormal fluctuations. To further quantify its dynamic behavior, a numerical step-response simulation was performed for the PI/Al_2_O_3_/NiCr–NiSi/SiO multilayer structure. The simulated thermal time constant and curve-extracted 90% response time were 0.0343 s and 0.0803 s, respectively, under the specified boundary conditions. Owing to its small thickness, low thermal mass, and good mechanical flexibility, the proposed thin-film thermocouple can be conformally attached to compact and curved electronic surfaces, indicating promising potential for real-time localized temperature monitoring of telecommunication equipment and other compact electronic systems.

## 1. Introduction

Modern telecommunication infrastructure, including base stations and communication cabinets, operates continuously with increasing integration density and power consumption [1]. The compact integration of electronic components inevitably leads to significant heat generation and temperature gradients within communication equipment, posing major challenges to the reliability and stable operation of telecommunication systems [2]. Previous studies have shown that excessive heat flux and non-uniform temperature distribution may produce localized hot spots and accelerate the degradation of electronic components, thereby reducing the reliability and service lifetime of electronic devices [3]. In practical telecommunication base stations, thermal conditions are influenced not only by internal electronic heat generation but also by external environmental factors such as ambient temperature and airflow, resulting in dynamic temperature variations during operation [4]. Consequently, continuous monitoring of thermal conditions is essential for maintaining equipment within safe operating temperature ranges and for supporting efficient thermal management strategies in telecommunication infrastructure.

With the development of intelligent operation and maintenance technologies in telecommunication networks, environmental monitoring systems have become an important component for ensuring the stable operation of communication infrastructure [5]. Such systems typically rely on distributed sensors to continuously collect environmental parameters such as temperature, humidity, and airflow within communication equipment and machine rooms [6]. The development of Internet of Things (IoT) and wireless sensor network technologies has facilitated the deployment of monitoring devices for real-time environmental sensing and data transmission in telecommunication facilities [7]. Wireless sensor networks provide an efficient framework for integrating sensing, communication, and data processing in environmental monitoring applications [8]. These technologies have been widely adopted in industrial facilities and smart infrastructure to support condition monitoring and fault detection [9]. However, conventional temperature sensing devices used in many monitoring systems are typically rigid, relatively large in size, and may exhibit limited response speed when applied to compact electronic structures with localized heat generation [10]. Therefore, the development of miniature, high-sensitivity, and flexible temperature sensors has become an important research direction for improving thermal monitoring capability in modern electronic systems [11].

To overcome the limitations of conventional temperature sensors, thin-film thermocouples (TFTCs) have attracted increasing attention for temperature monitoring in highly integrated electronic systems due to their small size, minimal disturbance to the measured surface, and capability for in situ surface temperature measurement [10]. In addition, the thin-film structure enables high spatial and temporal resolution, making TFTCs suitable for microscale thermal diagnostics and transient temperature measurement [12,13]. Thin-film thermocouples fabricated by magnetron sputtering have been reported to exhibit good linearity and stable thermoelectric performance, indicating the feasibility of sputtering-based fabrication for temperature sensing applications [14]. Recent studies have further demonstrated the development of film-based thermocouple sensors for harsh-environment and high-temperature applications. Hai et al. developed an Ag/Pt thick-film thermocouple using a mask-printing process for extreme elevated-temperature environments, highlighting the importance of structural stability and high-temperature durability in film-based thermocouple design [15]. Hu et al. reported a micro-nodal tungsten–rhenium thin-film thermocouple fabricated by electrohydrodynamic printing, showing millisecond-level dynamic response and good high-temperature measurement performance [16]. These studies indicate that material selection, film structure, fabrication method, junction size, and interfacial stability are important factors affecting thermocouple stability, measurement accuracy, and dynamic response.

Despite these advances, most existing studies mainly focus on high-temperature or harsh-environment applications, such as aerospace, energy, and extreme thermal-field measurement. In contrast, temperature monitoring in telecommunication equipment requires flexible, compact, low-cost, and conformally attachable sensors for localized monitoring on electronic modules, heat sinks, power devices, and communication cabinets. Therefore, the development of flexible NiCr–NiSi thin-film thermocouples on PI substrates remains meaningful for moderate-temperature telecommunication scenarios, where mechanical flexibility, local attachment, stable thermoelectric output, and compatibility with distributed monitoring are particularly important.

In this study, a flexible thin-film thermocouple sensor based on NiCr–NiSi thermoelectric materials was developed for temperature monitoring of telecommunication equipment. This work focuses not only on sensor fabrication but also on communication-oriented structural design and application-related performance evaluation. A multilayer PI/Al_2_O_3_/NiCr–NiSi/SiO structure was adopted to combine mechanical flexibility, electrical insulation, environmental protection, and stable thermoelectric output. Static calibration experiments and representative transient thermal tests were carried out to evaluate the sensitivity, repeatability, and dynamic temperature-tracking capability of the fabricated sensor. The results show that the proposed sensor exhibits stable temperature measurement performance and potential for real-time localized thermal monitoring of telecommunication equipment and other compact electronic systems.

## 2. Materials and Methods

### 2.1. Technical Principles of Thin-Film Thermocouple Temperature Sensors

Thin-film thermocouple sensors operate based on the Seebeck effect, which describes the generation of an electromotive force when a temperature difference exists between two junctions composed of dissimilar conductive materials. By measuring the thermoelectric voltage generated in the circuit, the temperature difference between the hot and cold junctions can be determined [17].

For a thermocouple composed of materials A and B, the thermoelectric potential can be expressed as

(1) $$E_{AB}=\left(S_{B}-S_{A}\right)\left(T_{1}-T_{2}\right)=S_{AB}\left(T_{1}-T_{2}\right)$$

where $E_{AB}$ is the thermoelectric voltage, $S_{A}$ and $S_{B}$ are the absolute Seebeck coefficients of materials *A* and *B*, respectively, and $T_{1}$ and $T_{2}$ denote the temperatures of the hot and reference junctions. Thus, the thermoelectric voltage is proportional to the temperature difference between the two junctions.

Compared with conventional wire thermocouples, thin-film thermocouples are fabricated directly on the surface of the measured object using microfabrication techniques, resulting in extremely small thermal mass and rapid dynamic response. These characteristics make thin-film thermocouples particularly suitable for surface temperature monitoring in highly integrated electronic systems [10].

The thermal response time of a thin-film thermocouple is closely related to the thermal inertia of the sensing layer and can be approximately expressed as

(2) $$\tau =\frac{\rho cd^{2}}{k}$$

where τ is the thermal response time, ρ is the density of the sensing material, c is the specific heat capacity, k is the thermal conductivity, and d is the characteristic thickness of the sensing layer. According to this relationship, the response time is proportional to the square of the film thickness; therefore, reducing the thickness of the thermoelectric layer can significantly improve the response speed of the sensor [10].

**Figure 1:**
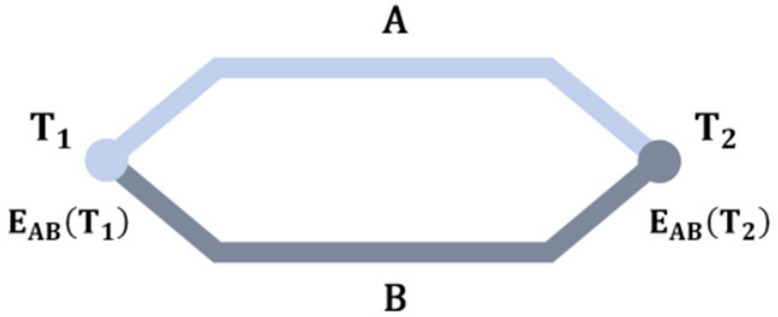
Schematic illustration of the Seebeck effect in a thermocouple, where A and B represent two dissimilar thermoelectric materials.

**Figure 2:**
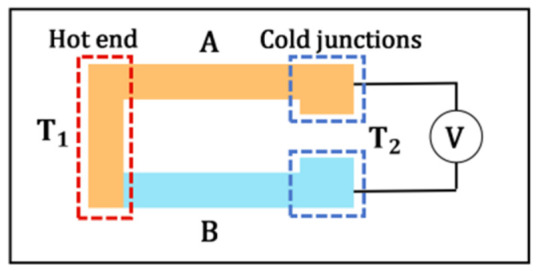
Schematic illustration of the operating principle of a thin-film thermocouple, where A and B represent two dissimilar thermoelectric materials.

Figure 1 and Figure 2 illustrate the basic structure and operating mechanism of the thin-film thermocouple used in this study. The hot junction is located at the intersection of two thin-film electrodes deposited on the substrate surface, while the cold junction is connected to the external measurement circuit. When a temperature gradient exists between the two junctions, the generated thermoelectric voltage can be converted into temperature using the thermoelectric calibration relationship.

Among various thermocouple materials, NiCr–NiSi thermocouples (K-type) provide a good balance between thermoelectric sensitivity, stability, and cost and are widely used in practical temperature monitoring applications [11]. In this study, NiCr and NiSi were adopted as the thermoelectric electrode materials.

### 2.2. Structural Design and Material Selection of the Thin-Film Thermocouple Sensor

Based on the working principle described above, the structural design of the proposed thin-film thermocouple sensor was determined according to the practical requirements of temperature monitoring of telecommunication equipment.

Outdoor telecommunication installations are often exposed to harsh environments. Ambient temperatures may vary from approximately −30 °C to 55 °C, while the internal temperature of communication cabinets can increase to about 65–85 °C because of solar radiation and heat generated by electronic modules [2]. Localized hot spots may also appear on processors, power modules, or heat sinks in densely integrated systems [3]. Therefore, sensors for telecommunication monitoring should provide moderate operating-temperature capability, high sensitivity, compact size, and relatively low cost for distributed deployment [6].

Previous studies have shown that thin-film thermocouple performance is strongly influenced by the thermoelectric material system, substrate structure, and electrode geometry, which together affect sensitivity, heat conduction, and dynamic response [10,18]. Accordingly, a multilayer flexible structure was designed to balance sensing performance with installation requirements.

**Figure 3:**
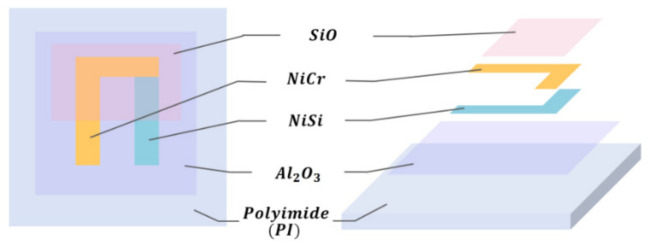
Multilayer structure of the proposed flexible thin-film thermocouple sensor.

The proposed sensor adopts a multilayer structure consisting of a polyimide (PI) substrate, an Al_2_O_3_ insulating layer, NiCr and NiSi thermoelectric layers, and a SiO protective layer, as illustrated in Figure 3.

The PI substrate provides mechanical flexibility for conformal attachment to curved or irregular surfaces and has been widely used in thin-film thermocouples for surface temperature measurement [10]. The Al_2_O_3_ layer electrically isolates the thermoelectric films from the substrate, while the SiO layer protects the films from oxidation and mechanical damage. Such oxide layers are commonly used to improve dielectric reliability and environmental durability [12].

The thermoelectric electrodes were formed by NiCr and NiSi thin films, corresponding to a K-type thermocouple pair. According to the thermoelectric relation described in Equation (1), the thermoelectric sensitivity of a thermocouple can be expressed as

(3) $$\frac{dE}{dT}=S_{AB}$$

where $S_{AB}$ represents the relative Seebeck coefficient between the two thermoelectric materials. Materials with larger Seebeck coefficients can generate higher thermoelectric voltage outputs under the same temperature difference [17]. Among commonly used thermocouple materials, noble-metal thermocouples such as PtRh–Pt provide excellent stability at extremely high temperatures but involve significantly higher material costs. In contrast, NiCr–NiSi thermocouples provide a favorable balance between thermoelectric sensitivity, stability, and cost, which explains their widespread application in industrial temperature measurement systems [17]. Considering that the operating temperature range of telecommunication equipment typically lies below 100 °C, the NiCr–NiSi thermocouple pair provides sufficient sensitivity and reliability while enabling cost-effective large-scale deployment.

The thicknesses of the NiCr and NiSi functional films were both set to 1000 nm. According to Equation (2), reducing film thickness helps improve response speed, whereas excessively thin films may reduce film continuity and electrical stability. Reported thermoelectric layers usually range from several hundred nanometers to several micrometers [13]; therefore, 1000 nm was selected to balance conductivity, structural reliability, and rapid response.

Sufficient film thickness and continuous thermoelectric layers help maintain stable electrical conduction paths and repeatable thermoelectric output. Therefore, the layer thickness and multilayer configuration were selected by considering film continuity, interfacial stability, and thermal transport across the sensor stack.

The Al_2_O_3_ insulating layer and SiO protective layer were both set to 600 nm. Thinner layers may cause leakage, whereas thicker layers increase thermal resistance and can degrade dynamic response. Submicrometer oxide layers are therefore commonly adopted for insulation and protection in thin-film thermocouple structures [12].

**Figure 4:**
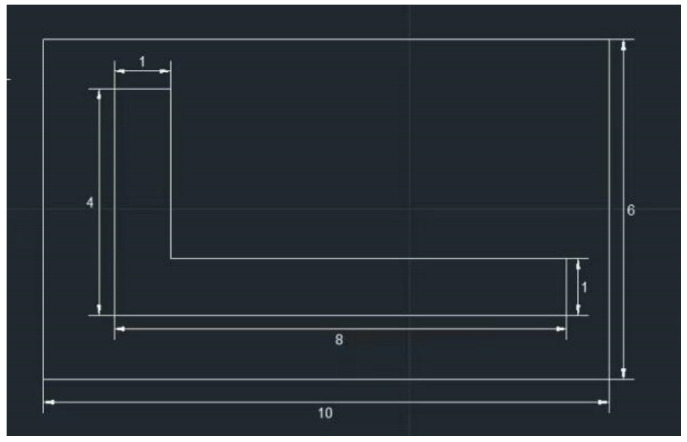
Geometric layout and key dimensions of the thin-film thermoelectric electrodes.

The planar geometry of the thermoelectric films was designed as an L-shaped layout, as illustrated in Figure 4. Each electrode consists of a vertical arm 1 mm wide and 4 mm high and a horizontal arm 8 mm long and 1 mm wide. A 1 mm gap was reserved at the corner region, and the overall sensor footprint was 10 mm × 6 mm. This layout was selected by considering electrical resistance, fabrication feasibility, and installation space in compact telecommunication equipment.

The electrical resistance of a thin-film conductor can be expressed as

(4) $$R=\rho_{e}\frac{L}{A}$$

where $\rho_{e}$ is the electrical resistivity, L is the conductor length, and A is the cross-sectional area. Increasing the electrode length facilitates signal extraction and external connection, but excessive lead conduction may disturb the local temperature field and introduce measurement errors [18].

The electrode width was set to 1 mm to ensure fabrication consistency and stable signal transmission. This millimeter-scale width is compatible with mask-based fabrication and helps avoid excessive resistance in the leads [19]. The overall sensor size was limited to 10 mm × 6 mm so that the device could be installed on densely integrated electronic components without significantly disturbing the thermal field [20].

Based on these considerations, the proposed multilayer structure and geometric dimensions represent a compromise among thermoelectric sensitivity, response speed, insulation reliability, material cost, and installation feasibility. The rationality of this design was further evaluated by numerical simulation.

### 2.3. Numerical Simulation Analysis of Thin-Film Thermocouple Performance

#### 2.3.1. Thermoelectric and Structural Simulation

To verify the structural design and material selection described in Section 2.2, numerical simulations were conducted in MATLAB R2024a (MathWorks, Natick, MA, USA) to analyze the thermoelectric behavior of the thin-film thermocouple under different conditions. The temperature range was extended from −200 °C to 1500 °C to cover both practical operating conditions and the broader characteristics of different thermocouple material systems.

**Figure 5:**
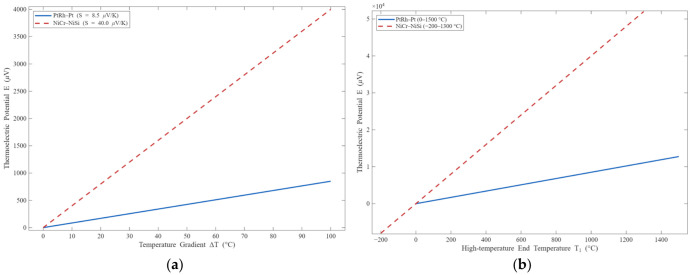
Comparison of thermoelectric potential over different temperature ranges. (**a**) Basic temperature range (0–100 °C), (**b**) Full temperature range.

**Figure 6:**
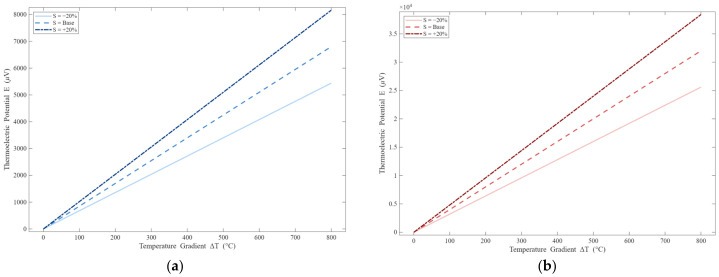
Effect of Seebeck coefficient variation (±20%) on thermoelectric potential. (**a**) PtRh–Pt, (**b**) NiCr–NiSi.

**Figure 7:**
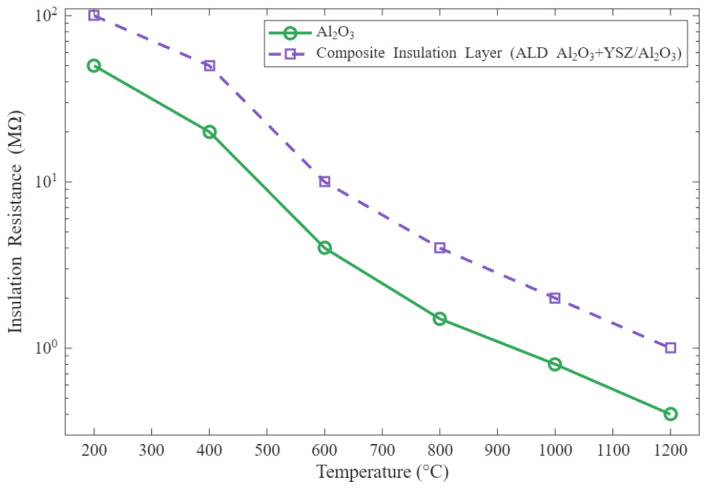
Resistance–temperature characteristics of different insulation structures.

**Figure 8:**
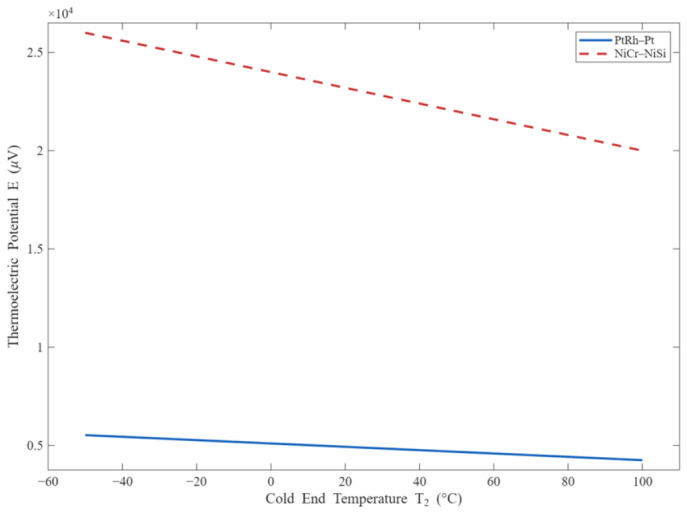
Effect of cold-junction temperature on thermoelectric potential ($T_{1}$ = 600 °C).

**Figure 9:**
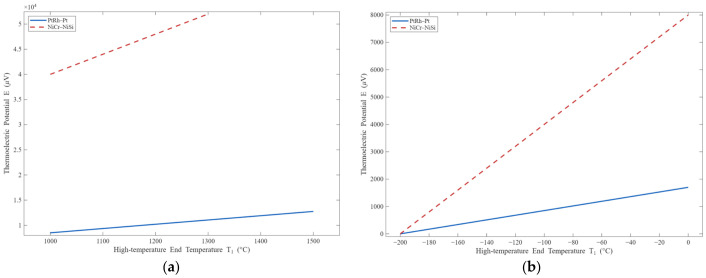
Thermoelectric potential responses of PtRh–Pt and NiCr–NiSi thermocouples under extreme temperature ranges. (**a**) High temperature range (1000–1500 °C), (**b**) Low temperature range (−200–0 °C).

**Figure 10:**
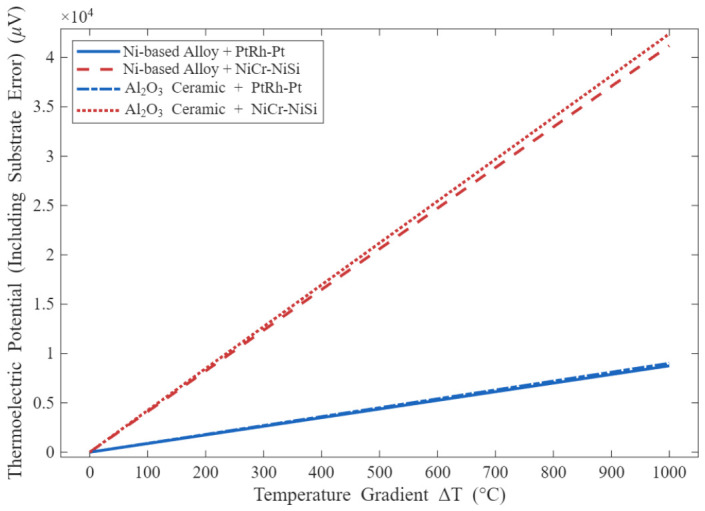
Effect of different substrate materials on thermoelectric potential (0–1000 °C).

**Figure 11:**
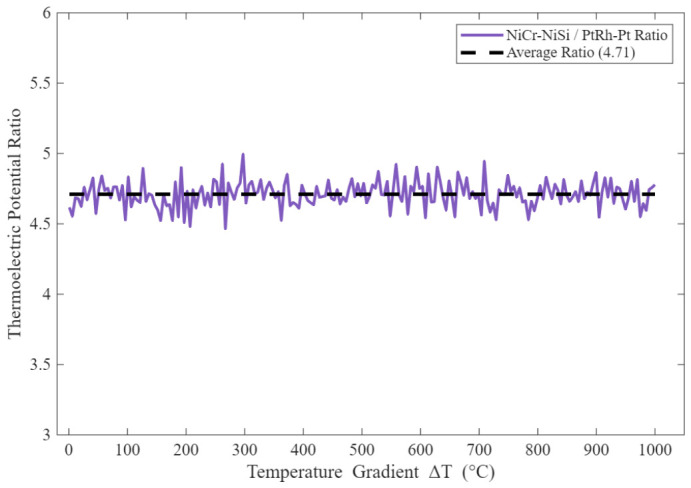
Relationship between the thermoelectric potential ratio of two materials and temperature.

Figure 5, Figure 6, Figure 7, Figure 8, Figure 9, Figure 10 and Figure 11 summarize the simulated effects of material sensitivity, Seebeck coefficient variation, insulation performance, cold-junction temperature, extreme-temperature response, substrate compatibility, and thermoelectric sensitivity ratio.

According to Equation (1), the thermoelectric voltage of a thermocouple is proportional to the temperature difference between the hot and cold junctions and depends on the Seebeck coefficients of the two materials. Simulations first compared the thermoelectric sensitivities of PtRh–Pt and NiCr–NiSi thermocouples. Figure 5 shows that within 0–100 °C, NiCr–NiSi exhibits higher thermoelectric sensitivity, whereas PtRh–Pt maintains better stability at extremely high temperatures when the range is extended to −200 °C to 1500 °C.

In practical thin-film thermocouples, variations in material composition or deposition conditions may change the effective Seebeck coefficient. The thermoelectric sensitivity can be defined as

(5) $$S=\frac{dE}{dT}$$

To evaluate this influence, the Seebeck coefficient was assumed to fluctuate within ±20% of its nominal value. The corresponding thermoelectric voltage can then be expressed as

(6) $$E=\left(S_{AB}+\Delta S\right)\left(T_{1}-T_{2}\right)$$

where ΔS represents the variation in the Seebeck coefficient caused by fabrication uncertainty or compositional deviation. Figure 6 presents the simulated thermoelectric potentials of PtRh–Pt and NiCr–NiSi thermocouples under ±20% Seebeck coefficient variation. The simulation results indicate that the thermoelectric voltage maintains an approximately linear relationship with temperature even when parameter fluctuations are introduced.

In thin-film thermocouples, the insulating layer electrically isolates the thermoelectric films from the substrate and prevents leakage currents that may distort thermoelectric measurements. The temperature dependence of insulation resistance can be described by the Arrhenius relation

(7) $$R(T)=R_{0}e^{E_{a}/kT}$$

where $R_{0}$ is the initial resistance, $E_{a}$ is the activation energy of electrical conduction, k is the Boltzmann constant, and T is the absolute temperature. Figure 7 compares the resistance–temperature characteristics of different insulation structures. As temperature increases, the insulation resistance decreases due to thermally activated charge transport, while multilayer insulation structures maintain higher resistance values and provide improved electrical isolation.

The thermoelectric voltage also depends on the cold-junction temperature. When the cold-junction temperature varies, the thermoelectric voltage can be expressed as

(8) $$E=S_{AB}\left(T_{hot}-T_{cold}\right)$$

Figure 8 shows the variation in thermoelectric potential when the hot-junction temperature is fixed at 600 °C and the cold-junction temperature changes from −50 °C to 100 °C. The thermoelectric voltage decreases as the cold-junction temperature increases because the effective temperature difference between the junctions becomes smaller.

When the temperature range becomes very wide, the Seebeck coefficient may vary with temperature. In this case, the thermoelectric voltage can be expressed as

(9) $$E=\int_{T_{2}}^{T_{1}}S(T)dT$$

where $S\left(T\right)$ represents the temperature-dependent Seebeck coefficient. Figure 9 illustrates the thermoelectric responses of PtRh–Pt and NiCr–NiSi thermocouples under extreme-temperature conditions. The simulation results show that both thermocouple materials maintain approximately linear thermoelectric behavior over a wide temperature range.

Thermal expansion mismatch between the substrate and thin-film layers may introduce interfacial stress and slightly affect the electrical resistance and Seebeck coefficient of the films. The thermal strain caused by expansion mismatch can be expressed as

(10) $$\epsilon =\left(\alpha_{s}-\alpha_{f}\right)\Delta T$$

where $\alpha_{s}$ and $\alpha_{f}$ represent the thermal expansion coefficients of the substrate and thin-film materials, respectively. Figure 10 compares the thermoelectric potentials obtained using different substrate materials. The results indicate that a larger thermal expansion mismatch may introduce deviations in thermoelectric output due to interfacial stress between the substrate and thin-film layers.

To further evaluate the relative thermoelectric performance of different materials, the ratio between the thermoelectric potentials of NiCr–NiSi and PtRh–Pt thermocouples can be expressed as

(11) $$\frac{E_{NiCr-NiSi}}{E_{PtRh-Pt}}=\frac{S_{NiCr-NiSi}}{S_{PtRh-Pt}}$$

Figure 11 shows the variation in the thermoelectric potential ratio with temperature. The ratio remains within a relatively stable range and is consistent with the theoretical ratio derived from the corresponding Seebeck coefficients.

Overall, the numerical simulations verify the thermoelectric characteristics, insulation performance, and structural rationality of the proposed thin-film thermocouple design.

#### 2.3.2. Numerical Step-Response Simulation

To further quantify the dynamic response behavior of the proposed sensor, a numerical step-response simulation was performed using a one-dimensional multilayer thermal resistance-capacitance (RC) model. The simulated structure consisted of a PI substrate, an Al_2_O_3_ insulating layer, an equivalent NiCr–NiSi thermoelectric layer, and a SiO protective layer, consistent with the designed PI/Al_2_O_3_/NiCr–NiSi/SiO sensor structure. The thicknesses of the NiCr–NiSi functional layer, Al_2_O_3_ insulating layer, and SiO protective layer were set according to the fabricated sensor design, while the PI substrate thickness was set to 50 μm in the baseline simulation.

The initial temperature of the multilayer structure was set to 25 °C. A step thermal excitation from 25 °C to 85 °C was applied to the bottom surface of the PI substrate, and the upper SiO surface was exposed to natural convection with ambient air at 25 °C. The equivalent contact heat-transfer coefficient between the monitored surface and the PI substrate was set to 5000 W/(m^2^·K), and the natural convection coefficient was set to 10 W/(m^2^·K). The NiCr–NiSi thermoelectric layer was selected as the sensing-junction node, and its transient temperature was extracted as the simulated sensor response.

(12) $$T(t)=T_{\infty }-\left(T_{\infty }-T_{0}\right)e^{-t/\tau }$$

where T(t) is the sensing-junction temperature at time t, $T_{0}$ is the initial temperature, $T_{\infty }$ is the steady-state temperature, and τ is the thermal time constant. The 90% response time was calculated as $t_{90}$ = 2.303τ and was also directly extracted from the normalized temperature response curve.

**Figure 12:**
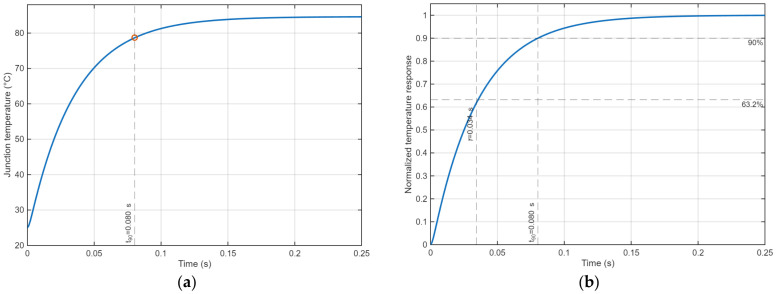
Simulated step response under a 25–85 °C temperature step. (**a**) Sensing-junction temperature response, (**b**) Normalized response for extracting the thermal time constant and 90% response time.

Figure 12 shows the simulated step-response characteristics of the proposed PI/Al_2_O_3_/NiCr–NiSi/SiO thin-film thermocouple under the temperature step from 25 °C to 85 °C. The sensing-junction temperature increased rapidly after the step thermal excitation and gradually approached the steady-state value. The normalized response curve was used to extract the thermal time constant and 90% response time.

By fitting the normalized response using the first-order thermal response model, the thermal time constant τ was estimated to be 0.0343 s, and the corresponding fitted $t_{90}$ response time was 0.0790 s. The directly extracted t90 from the simulated response curve was 0.0803 s, which is consistent with the fitted result. This agreement indicates that the dynamic behavior of the multilayer sensing structure can be reasonably approximated by a first-order thermal response under the specified boundary conditions.

Considering that the flexible PI substrate contributes significantly to the thermal inertia of the multilayer structure, a parametric simulation was further conducted by varying the PI substrate thickness from 25 μm to 100 μm while keeping the other structural and boundary parameters unchanged. The simulated dynamic response indicators are summarized in Table 1.

**Figure 13:**
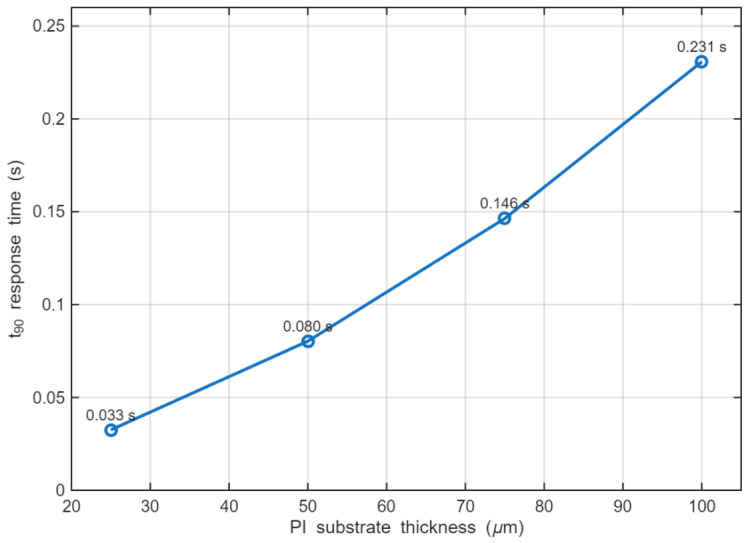
Effect of PI substrate thickness on the simulated t90 response time.

As shown in Table 1, the simulated response time increased with the PI substrate thickness. To more clearly show this trend, the curve-extracted $t_{90}$ values were plotted as a function of PI substrate thickness, as shown in Figure 13.

As shown in Figure 13, the simulated $t_{90}$ increased from 0.0325 s to 0.2308 s when the PI substrate thickness increased from 25 μm to 100 μm. This trend indicates that the substrate thermal inertia plays an important role in the overall dynamic response of the flexible thin-film thermocouple. Therefore, reducing substrate thickness, improving thermal contact, or optimizing the multilayer structure may further enhance the dynamic response of the sensor.

The simulated thermoelectric voltage response was also calculated using the measured sensitivity of 40.45 μV/°C. Since the thermoelectric voltage is linearly related to the temperature difference between the hot and cold junctions, the voltage response follows the same dynamic trend as the sensing-junction temperature. Therefore, only the temperature response and normalized response are presented here to avoid repeated information.

It should be noted that the above response indicators were obtained from numerical simulation rather than direct experimental step-response calibration. Therefore, the extracted τ and $t_{90}$ values should be interpreted as estimated dynamic indicators under the specified thermal boundary conditions. Direct step-heating experiments will be conducted in future work to further validate the simulated response time.

### 2.4. Experimental Procedures

#### 2.4.1. Fabrication of the Flexible Thin-Film Thermocouple

Flexible thin-film thermocouple sensors were fabricated on polyimide (PI) substrates according to the structural design described in Section 2.2. The multilayer sensor consisted of a PI substrate, an Al_2_O_3_ insulating layer, NiCr and NiSi thermoelectric films, and a SiO protective layer. PI is widely used in flexible thin-film thermocouple devices because of its good mechanical flexibility, thermal resistance, and electrical insulation properties [10]. Prior to deposition, the PI substrates were cut to the designed dimensions, ultrasonically cleaned in acetone, ethanol, and deionized water, dried with nitrogen, and mildly heated to remove residual moisture. Such pretreatment is commonly used to improve surface cleanliness, interfacial adhesion, and deposition quality in thin-film fabrication [12]. Film deposition was carried out by physical vapor deposition (PVD) in a magnetron sputtering system, which is commonly used for preparing functional thin-film structures [21]. Before deposition, the chamber was evacuated to about 5 × 10^−4^ Pa; during deposition, the substrate was kept at room temperature, the holder rotation speed was about 5 rpm, and the target-to-substrate distance was 10 cm.

The Al_2_O_3_ insulating layer was first deposited by radio-frequency magnetron sputtering using a high-purity Al_2_O_3_ target at an Ar flow rate of 30 sccm, a sputtering power of 180 W, a working pressure of 0.35 Pa, and a deposition rate of about 0.2 Å/s. Al_2_O_3_ thin films are widely used as insulating layers in thin-film sensors because of their good dielectric properties and thermal stability [22]. The NiCr thermoelectric film was then deposited by direct-current magnetron sputtering using a high-purity NiCr target at 30 sccm Ar, 90 W, 0.35 Pa, and about 0.8 Å/s. The NiSi thermoelectric film was deposited by direct-current magnetron sputtering at 70 W, 0.4 Pa, and about 0.5 Å/s. NiCr–NiSi is a commonly used thermoelectric material combination for thin-film thermocouples because of its relatively high sensitivity and stable thermoelectric performance [22]. Mask-assisted deposition was used to define the thermoelectric electrodes, which were arranged in an L-shaped configuration to form the sensing junction. According to the design in Section 2.2, the electrode width was about 1 mm, and the sensing area was about 10 mm × 6 mm. Finally, a SiO protective layer was deposited by radio-frequency sputtering at 120 W, 0.2 Pa, and about 0.3 Å/s, and external lead wires were connected with conductive silver paste before stabilization and testing. Oxide-based protective layers are commonly used to improve the environmental stability of thin-film thermocouple devices [23].

These deposition and pretreatment procedures may affect film continuity, interfacial adhesion, residual stress, and environmental stability. In this work, the multilayer structure was controlled through target materials, sputtering parameters, deposition sequence, deposition rate, and mask-defined electrode geometry. Further structural and compositional characterization is still needed to verify film morphology, composition uniformity, and interface quality.

#### 2.4.2. Static Calibration and Characterization

**Figure 14:**
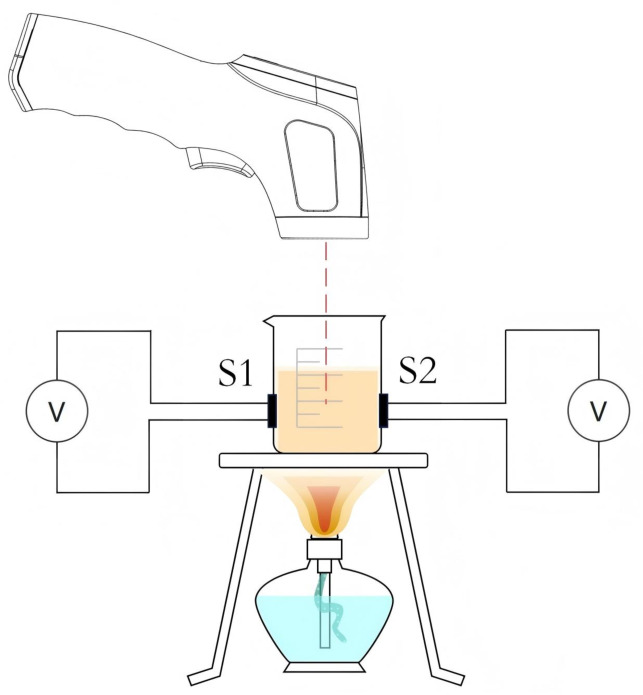
Schematic diagram of the static calibration and characterization setup.

Static calibration experiments were conducted to evaluate the thermoelectric response characteristics of both a commercial thermocouple and the fabricated thin-film thermocouple. The experimental setup is illustrated in Figure 14.

The setup consisted of a glass container filled with heat-transfer oil, an alcohol lamp used as the heating source, an infrared thermometer for monitoring the heating condition, and a digital multimeter for measuring thermoelectric voltage. Heat-transfer oil was selected as the heating medium because it provides a relatively uniform thermal field during heating.

First, a commercial K-type thermocouple was used to verify the stability of the measurement system and provide a reference thermoelectric response. The thermocouple probe was immersed in the heated oil while the generated thermoelectric voltage was recorded using the digital multimeter.

After verifying the measurement system, the fabricated thin-film thermocouple sensor was tested using the same experimental setup. The thermoelectric junction was exposed to the heating environment, and the corresponding thermoelectric voltage output was recorded as the temperature increased. To evaluate measurement repeatability and stability, six heating–cooling cycles were performed. At each temperature stage, the thermoelectric voltage was recorded after the thermal condition became stable. The collected voltage–temperature data were used to analyze the thermoelectric sensitivity and linear response characteristics of the fabricated sensor, which are important parameters for evaluating thin-film thermocouple performance.

In addition to sensitivity and linearity analysis, the main measurement uncertainty sources were considered during calibration and data interpretation. For thermocouple-based temperature measurement, the hot-junction temperature can be expressed as:

(13) $$T_{h}=T_{c}+\frac{E}{S}$$

where $T_{h}$ is the hot-junction temperature, $T_{c}$ is the cold-junction temperature, E is the measured thermoelectric voltage, and S is the calibrated thermoelectric sensitivity. Therefore, cold-junction temperature fluctuation, voltage measurement uncertainty, calibration fitting error, reference temperature uncertainty, lead heat conduction, and spatial temperature gradients may contribute to the final temperature measurement error. The combined temperature uncertainty was estimated using the root-sum-square method:

(14) $$u_{T}=\sqrt{u_{cold}^{2}+{\left(\frac{u_{E}}{S}\right)}^{2}+u_{fit}^{2}+u_{ref}^{2}+u_{lead}^{2}+u_{spatial}^{2}}$$

where $u_{cold}$, $u_{E}/S$, $u_{fit}$, $u_{ref}$, $u_{lead}$ and $u_{spatial}$ represent the uncertainty contributions from cold-junction temperature variation, voltage measurement, calibration fitting, reference temperature measurement, lead heat conduction, and spatial temperature difference, respectively.

#### 2.4.3. Dynamic Performance and Environmental Adaptability Test

**Figure 15:**
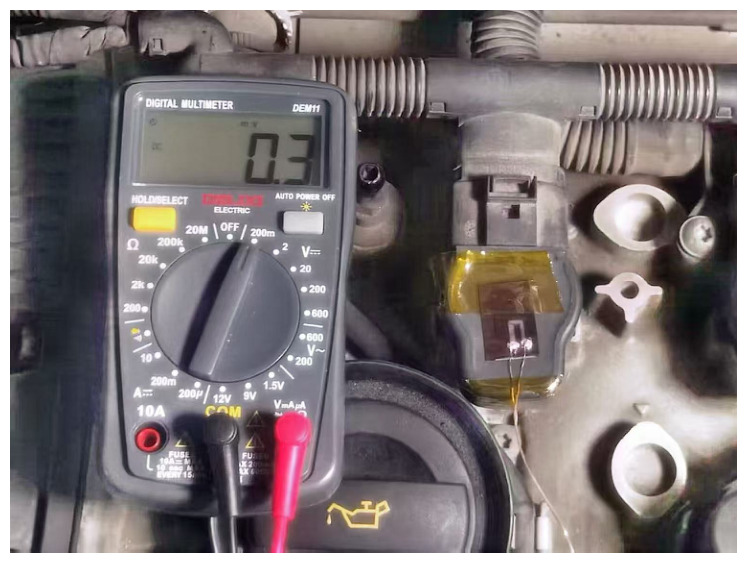
Dynamic temperature measurement setup under transient thermal conditions.

To further evaluate the dynamic temperature measurement capability of the fabricated sensor, transient temperature measurements were carried out under representative engineering operating conditions. The test configuration generated continuous temperature variations caused by changes in thermal load, airflow, and heat dissipation, thus providing representative heating and cooling transients relevant to electronic temperature monitoring. The dynamic temperature measurement setup is shown in Figure 15.

During the experiment, the sensing junction and the reference thermocouple were placed at adjacent positions on the monitored surface to minimize spatial temperature differences. The thermoelectric voltage generated by the thin-film thermocouple was recorded continuously and converted to temperature using the static calibration relationship obtained in Section 3.1. The corresponding thermal histories at the measurement location were then analyzed under several typical transient operating states.

The measured processes included temperature rise under relatively weak convective cooling, temperature rise under elevated thermal load, and temperature decrease under enhanced cooling conditions. These representative transients were used to assess whether the fabricated thin-film thermocouple could maintain stable output and continuous temperature tracking under non-steady thermal conditions.

Such dynamic tests are commonly used to evaluate the response performance and practical applicability of thin-film thermocouple sensors in real engineering environments [24].

## 3. Results

### 3.1. Static Calibration Results

The thermoelectric characteristics of commercial K-type thermocouples and the fabricated thin-film thermocouple were first evaluated through static calibration experiments.

**Figure 16:**
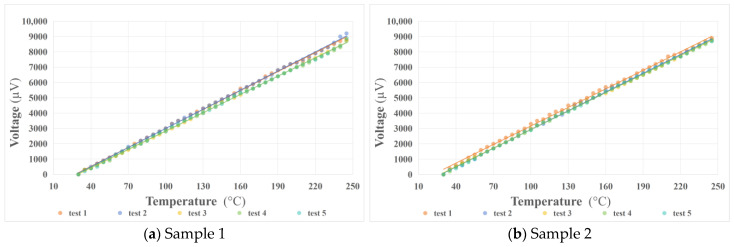
Thermoelectric calibration curves of two commercial K-type thermocouples.

Figure 16 shows the temperature–thermoelectric voltage relationships obtained from two commercial K-type thermocouples during repeated heating and cooling cycles. In both samples, the thermoelectric voltage increased approximately linearly with temperature over the investigated range, demonstrating good linear thermoelectric behavior.

The fitted slopes of Sample 1 ranged from 39.9 to 41.4 µV/°C, while those of Sample 2 ranged from 39.7 to 40.7 µV/°C. The standard deviations of the slopes for both samples were below 1 µV/°C, demonstrating good repeatability of the measurement system.

**Figure 17:**
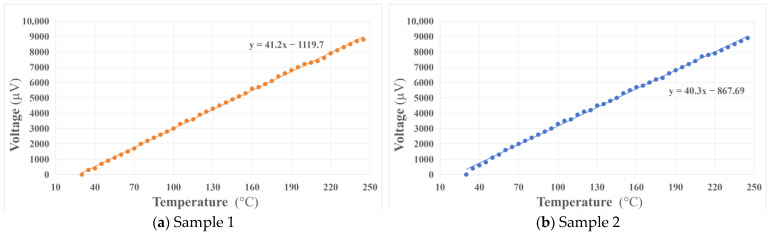
Representative linear fitting results of the commercial K-type thermocouples.

**Figure 18:**
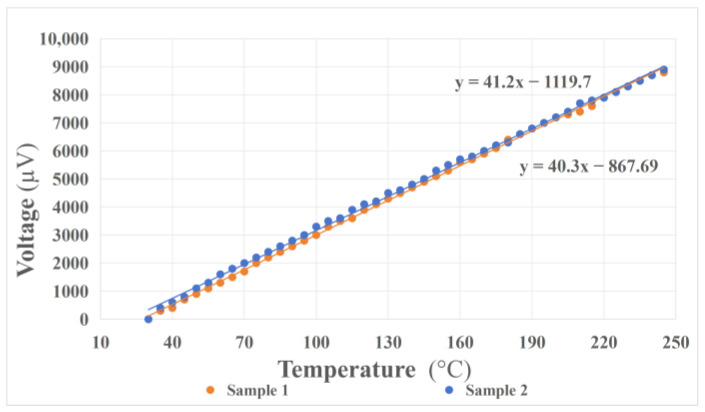
Comparison of representative thermoelectric calibration curves of the two commercial K-type thermocouples.

Representative linear fitting curves are presented in Figure 17, and the direct comparison between the two commercial thermocouples is illustrated in Figure 18. The fitted slope of Sample 1 was 41.2 µV/°C, corresponding to a relative error of 0.49% with respect to the reference sensitivity of 41 µV/°C defined in GB/T 16839.1-2018 [25]. In contrast, Sample 2 exhibited a slope of 40.3 µV/°C, with a relative error of −1.71%. Both results fall within the allowable tolerance range of the standard.

After verifying the reliability of the experimental system, the fabricated thin-film thermocouple was calibrated using the same experimental setup. The use of identical calibration conditions enables a direct comparison between the fabricated sensor and the commercial thermocouples, while also reducing the influence of experimental uncertainty. This provides a reliable basis for further evaluating the linearity, sensitivity, and repeatability of the fabricated sensor.

**Figure 19:**
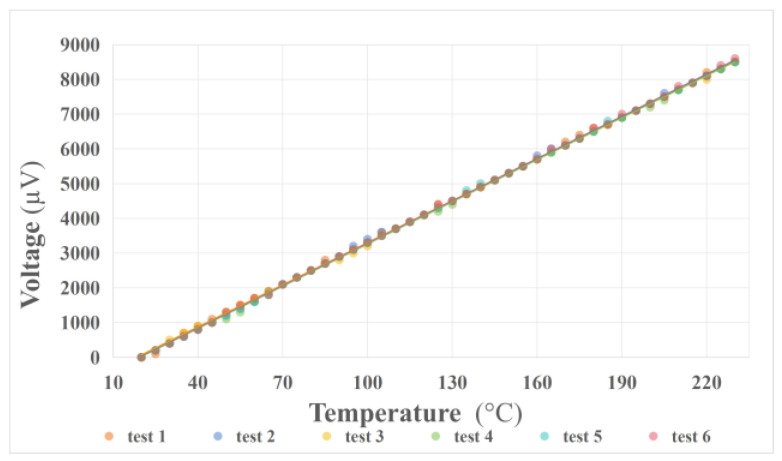
Temperature–thermoelectric voltage curves of the thin-film thermocouple.

Figure 19 shows the temperature–thermoelectric voltage curves obtained from six independent heating–cooling cycles. In all cases, the thermoelectric voltage increased monotonically with temperature and exhibited an approximately linear relationship across the tested temperature range. Such linear thermoelectric behavior is consistent with the typical response characteristics reported for thin-film thermocouples based on NiCr–NiSi materials.

The extracted slopes ranged from 40.2 to 40.6 µV/°C, indicating a highly consistent thermoelectric response under repeated thermal cycling. The fitted slopes and corresponding relative errors are summarized in Table 2.

The average slope was calculated as 40.45 µV/°C, with a standard deviation of 0.14 µV/°C, indicating good repeatability of the fabricated sensor. Using the standard reference sensitivity of 41 µV/°C, the relative error of the average slope was approximately 1.34%, and all six measurements exhibited deviations within ±2% of the reference value.

**Figure 20:**
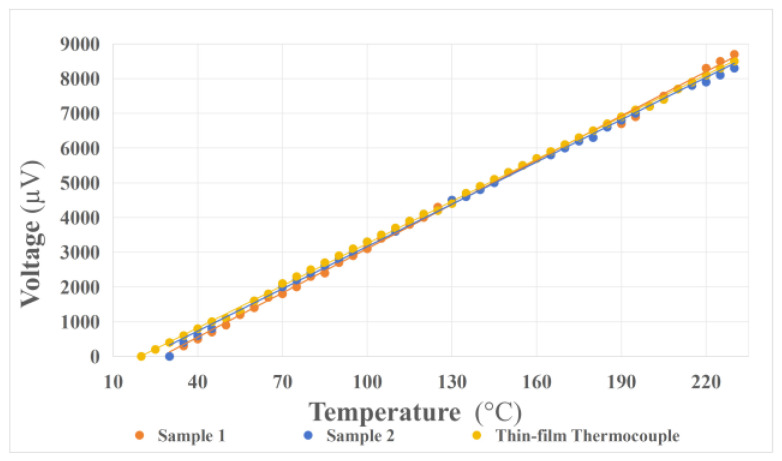
Comparison of temperature–thermoelectric potential curves of the thin-film thermocouple and commercial K-type thermocouples.

To further evaluate the performance of the fabricated sensor, its thermoelectric response was compared with that of the two commercial thermocouples. This comparison helps determine whether the fabricated thin-film thermocouple exhibits thermoelectric sensitivity comparable to that of conventional K-type thermocouples under the same calibration conditions. Figure 20 presents the comparison of temperature–thermoelectric potential curves between the fabricated thin-film thermocouple and the commercial sensors.

Table 3 summarizes the representative slopes and relative errors of the three thermocouples, providing a direct quantitative comparison between the fabricated thin-film sensor and the two commercial K-type thermocouples. The representative slope of the fabricated thin-film thermocouple was approximately 40.5 µV/°C, while those of the two commercial thermocouples were 41.2 µV/°C and 40.3 µV/°C, respectively. The relative differences between the fabricated sensor and the commercial samples were 1.70% and 0.50%, indicating comparable thermoelectric sensitivity to conventional K-type thermocouples.

Overall, the comparison indicates that the fabricated sensor exhibits a thermoelectric response level close to that of commercially available K-type thermocouples under the same calibration conditions.

### 3.2. Dynamic Temperature Response Under Real Operating Conditions

To evaluate the dynamic temperature measurement capability of the fabricated thin-film thermocouple, transient measurements were conducted under the representative conditions described in Section 2.4.3. These tests were used to evaluate the continuity and stability of the sensor output under time-varying thermal conditions.

**Figure 21:**
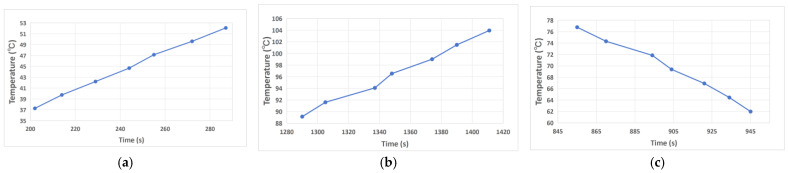
Dynamic temperature response of the thin-film thermocouple under different thermal conditions. (**a**) Temperature rise under low airflow conditions, (**b**) Temperature rise under increased thermal load, (**c**) Temperature decrease under enhanced cooling.

Figure 21 illustrates the temperature–time responses of the fabricated thin-film thermocouple under three representative thermal conditions: temperature rise under low airflow, temperature rise under elevated thermal load, and temperature decrease under enhanced cooling. These thermal processes represent typical transient heat-transfer behaviors relevant to compact electronic systems, such as communication cabinets and telecommunication base stations.

Under the first condition (Figure 21a), the measured temperature increased from approximately 37.3 °C to 52.1 °C within about 90 s, corresponding to an average heating rate of approximately 0.16 °C/s. Under the second condition (Figure 21b), the temperature increased from approximately 89.1 °C to 104.0 °C over approximately 140 s, corresponding to an average heating rate of approximately 0.11 °C/s. In contrast, under the third condition (Figure 21c), the measured temperature decreased from approximately 76.8 °C to 62.0 °C within about 100 s, corresponding to an average cooling rate of approximately −0.15 °C/s.

Across all operating conditions, the temperature curves remain smooth and continuous without observable signal interruption or abnormal fluctuations. The thermoelectric output varies consistently with the thermal conditions at the measurement location, indicating that the fabricated thin-film thermocouple can effectively track transient temperature variations under dynamic environments. Similar stable transient responses have also been reported for thin-film thermocouples used in rapid heat-transfer measurements and dynamic thermal monitoring applications [24]. Together with the extremely low thermal mass of the sensing junction, these results support the rapid-response capability of the proposed thin-film thermocouple.

## 4. Discussion

### 4.1. Static Thermoelectric Performance and Structural Advantages

The results presented in Section 3 show that the fabricated thin-film thermocouple exhibits stable static thermoelectric characteristics and stable dynamic tracking behavior under the tested thermal conditions.

In the static calibration experiments, both the commercial K-type thermocouples and the fabricated thin-film thermocouple showed highly linear voltage–temperature relationships. The fitted sensitivities remained within the tolerance range specified by GB/T 16839.1-2018, with relative errors below ±2%. The low dispersion of the fitted slopes during repeated heating–cooling cycles indicates good short-term repeatability, and the measured average sensitivity of 40.45 µV/°C is close to the nominal value of conventional K-type thermocouples.

Compared with conventional wire-type thermocouples, the fabricated thin-film device provides comparable thermoelectric sensitivity while offering the additional benefits of small thickness, mechanical flexibility, and conformal attachment to irregular surfaces. These features are advantageous for compact electronic systems with limited installation space, where conventional rigid or wire-type probes may disturb the local thermal field or be difficult to install.

Table 4 compares representative film-based thermocouple studies in terms of materials, fabrication method, substrate flexibility, application temperature, and main performance.

As shown in Table 4, recent film-based thermocouple studies mainly focus on high-temperature, fuel-cell, or rigid-substrate applications. In contrast, the present work focuses on moderate-temperature localized monitoring of telecommunication equipment using a flexible NiCr–NiSi thin-film thermocouple on a PI substrate. This comparison highlights the combined use of K-type thermoelectric materials, PI-based flexibility, conformal attachment, and telecom-oriented structural design.

### 4.2. Dynamic Response and Engineering Applicability

The dynamic experiments further show that the thin-film thermocouple can continuously capture transient temperature variations under changing thermal conditions. Across the tested conditions, the temperature curves remained smooth and continuous without observable signal interruption or abnormal fluctuation, suggesting stable output behavior under the representative transient thermal conditions.

The numerical step-response simulation in Section 2.3.2 further provides quantitative dynamic indicators for the proposed multilayer structure. Under the specified boundary conditions, the fitted thermal time constant τ was 0.0343 s, and the curve-extracted t90 response time was 0.0803 s. These simulated results support the rapid-response potential of the PI/Al_2_O_3_/NiCr–NiSi/SiO thin-film structure. The parametric simulation also shows that increasing the PI substrate thickness leads to a longer response time, indicating that substrate thermal inertia is an important factor affecting the dynamic behavior of the flexible thin-film thermocouple.

Although direct experimental step-response calibration was not performed in this work, the combination of representative transient tracking tests and numerical step-response analysis provides useful information for evaluating the dynamic behavior of the proposed sensor. The tested thermal processes are also relevant to compact electronic and communication systems. Temperature rise under reduced cooling is analogous to heat accumulation inside communication cabinets, while the other tested processes reflect changes in thermal load and convective heat dissipation. Therefore, the results provide useful engineering guidance for localized temperature monitoring of telecommunication equipment.

From an engineering perspective, flexible thin-film thermocouples can be directly attached to heat sinks, electronic chips, power modules, or communication cables, enabling localized temperature monitoring at critical positions where conventional probes are difficult to install. This installation advantage is particularly important for densely integrated electronic devices with limited available sensing space.

### 4.3. Measurement Error and Uncertainty Analysis

In addition to sensitivity, repeatability, and dynamic tracking behavior, measurement accuracy is affected by cold-junction temperature fluctuation, voltage measurement uncertainty, reference temperature uncertainty, calibration fitting error, lead heat conduction, and spatial temperature gradients. According to the thermocouple temperature–conversion relationship, the calculated hot-junction temperature depends on both the measured thermoelectric voltage and the cold-junction temperature. Therefore, cold-junction fluctuation directly introduces temperature error. In this work, the cold-junction side was kept under the same ambient condition during calibration and dynamic testing, and the commercial thermocouples and fabricated thin-film thermocouple were tested under identical calibration conditions. This arrangement reduces systematic deviation when comparing the fabricated sensor with commercial K-type thermocouples, but it does not completely eliminate the influence of cold-junction temperature fluctuation.

Lead heat conduction is another potential error source. The L-shaped electrode geometry and external lead wires may introduce additional heat conduction paths between the sensing junction and the external circuit. Excessive lead conduction can disturb the local temperature field near the sensing junction and cause deviation between the actual surface temperature and the measured thermoelectric response. To reduce this influence, the sensor footprint was limited to 10 mm × 6 mm, and the electrode width was set to 1 mm to balance electrical resistance, fabrication consistency, and thermal disturbance. During dynamic testing, the thin-film thermocouple and reference thermocouple were placed at adjacent positions to reduce spatial temperature differences.

The main error sources and their influence mechanisms are summarized in Table 5.

### 4.4. Microstructure–Interface–Property Relationship

The thermoelectric performance of thin-film thermocouples is closely related to film continuity, conductive paths, and interfacial bonding. In the PI/Al_2_O_3_/NiCr–NiSi/SiO multilayer structure, the NiCr and NiSi films form the functional thermoelectric layers, while the Al_2_O_3_ and SiO layers provide insulation and environmental protection. Therefore, the sensitivity and repeatability of the sensor are affected not only by the Seebeck coefficients of NiCr and NiSi but also by film continuity, interfacial adhesion, residual stress, and possible interfacial thermal resistance.

Continuous NiCr and NiSi films help maintain stable carrier transport and reduce local resistance fluctuation. The 1000 nm functional-film thickness was selected to balance electrical conductivity, film continuity, and low thermal mass. Stable interfaces among the PI substrate, Al_2_O_3_ insulating layer, thermoelectric films, and SiO protective layer may also help reduce delamination and contact degradation during heating–cooling cycles.

The calibration results, with an average sensitivity of 40.45 μV/°C and a standard deviation of 0.14 μV/°C, indicate stable thermoelectric output under the tested conditions. The step-response simulation further suggests that the dynamic behavior is affected by the thermal inertia of the multilayer structure. However, direct structural characterization by SEM, EDS, AFM, XRD, and cross-sectional analysis was not performed in this study. Therefore, this section provides a mechanism-based interpretation rather than direct microstructural evidence. Future work will include structural and compositional characterization to verify film morphology, composition uniformity, interface bonding, and residual stress.

### 4.5. Reliability Considerations and Limitations

The repeated heating–cooling calibration tests indicate good short-term repeatability of the fabricated thin-film thermocouple. The small dispersion of the fitted thermoelectric sensitivities across these cycles suggests stable output of the NiCr–NiSi films under the tested calibration conditions. However, because these tests involved only a limited number of cycles and did not include mechanical deformation, they should not be interpreted as verification of long-term durability or bending reliability.

From the structural-design perspective, the PI substrate provides mechanical flexibility for conformal attachment, while the Al_2_O_3_ insulating layer and SiO protective layer help improve electrical insulation and reduce environmental exposure of the thermoelectric films. These structural features are consistent with previous studies showing that flexible substrates and protective layers are important for mechanical adaptability and environmental stability [10,12]. Recent research on high-stability flexible thin-film temperature sensors has also shown that deformation during installation and stress during testing can affect sensor performance [26].

Nevertheless, long-term high-temperature aging, repeated thermal cycling, and bending-cycle tests under controlled bending radii were not performed in this study. The effects of cyclic mechanical deformation on resistance, sensitivity, and output drift remain to be evaluated. Therefore, the current results mainly demonstrate short-term repeatability and stable dynamic tracking behavior under the tested conditions, rather than complete long-term mechanical reliability. Future work will include long-term aging, thermal cycling, cyclic bending fatigue, and bending-radius-dependent output stability tests.

Overall, the fabricated thin-film thermocouple demonstrates accurate temperature measurement, good short-term repeatability, and stable dynamic tracking behavior under the tested conditions. Its thermoelectric sensitivity is close to the reference value of conventional K-type thermocouples, while the thin-film structure offers practical advantages including small thickness, flexibility, conformal attachment, and rapid-response potential. Further aging and bending-fatigue tests are still needed before large-scale engineering deployment.

## 5. Conclusions

In this study, a flexible NiCr–NiSi thin-film thermocouple sensor was developed for temperature monitoring of telecommunication equipment. By combining a multilayer PI/Al_2_O_3_/NiCr–NiSi/SiO structure with magnetron sputtering and microfabrication processes, the fabricated sensor exhibited good thermoelectric sensitivity, repeatability, and dynamic temperature-tracking capability. The static calibration results showed good linearity, and the measured sensitivity remained within the tolerance range specified by the national standard GB/T 16839.1-2018 [25]. The average sensitivity of the fabricated sensor was 40.45 µV/°C, which is close to the nominal value of standard K-type thermocouples and is in good agreement with the sensitivities of the two commercial K-type thermocouples used in this study.

Dynamic experiments under representative thermal conditions further showed that the thin-film thermocouple could continuously capture transient temperature variations without abnormal signal fluctuations, indicating good dynamic measurement capability. Owing to its small thickness, low thermal mass, and flexible structure, the proposed sensor is well-suited for localized temperature monitoring on complex electronic surfaces and shows strong potential for rapid-response thermal monitoring in telecommunication-related applications.

Future work will focus on direct step-response calibration, long-term aging, repeated thermal cycling, cyclic bending fatigue, bending-radius-dependent output stability tests, structural and compositional characterization, and integration with distributed monitoring systems for intelligent thermal monitoring in telecommunication infrastructure.

## Figures and Tables

**Table 1:** Simulated response time at different PI substrate thicknesses.

PI Thickness (μm)	τ (s)	Fitted $t_{90}$ (s)	Curve-Extracted $t_{90}$ (s)
25	0.0139	0.0319	0.0325
50	0.0343	0.0790	0.0803
75	0.0627	0.1444	0.1464
100	0.0991	0.2281	0.2308

**Table 2:** Fitted slopes and relative errors of the thin-film thermocouple.

Test No.	Slope (µV/°C)	Relative Error with Respect to the Reference Value (41 µV/°C) (%)
1	40.5	1.22
2	40.4	1.46
3	40.2	1.95
4	40.5	1.22
5	40.5	1.22
6	40.6	0.98

**Table 3:** Comparison of representative slopes and relative errors between the thin-film thermocouple and commercial K-type thermocouples.

Thermocouple Type	Representative Slope (µV/°C)	Relative Error with Respect to the National Standard (%)	Relative Error with Respect to the Representative Slope of the Thin-Film Thermocouple (%)
Thin-film thermocouple	40.5	1.22	–
Commercial sample 1 (S1)	41.2	0.49	1.70
Commercial sample 2 (S2)	40.3	−1.71	0.50

**Table 4:** Comparison of representative film-based thermocouple studies.

Study	Materials	Fabrication	Substrate/Flexibility	Temperature/Application	Main Performance
Luo et al. [14]	CuNi/TiB_2_	Magnetron sputtering	Dielectric substrate; rigid	Up to ~400 °C	Seebeck coefficient: 38.07 μV/°C
Hai et al. [15]	Ag/Pt	Mask printing	High-temperature substrate; rigid	900 °C for 50 h	Stability: 0.022%/h at 900 °C
Hu et al. [16]	W/Re	Electrohydrodynamic printing	100 μm alumina; rigid	300–1200 °C	Accuracy better than ±1.2%; response time: 1.2 ms
Liu et al. [22]	NiCr–NiSi	Magnetron sputtering	PI substrate; flexible	Fuel-cell thermal monitoring	Error within ±0.5 °C; response time < 15 μs
This work	NiCr–NiSi	Magnetron sputtering	PI substrate; flexible	Telecom equipment monitoring	Sensitivity: 40.45 μV/°C; simulated $t_{90}$ *: 0.0803 s

* $t_{90}$, 90% response time; the value in this work was obtained from numerical simulation.

**Table 5:** The main error sources affecting temperature measurement accuracy.

Error Source	Influence on Measurement	Evaluation or Mitigation Method
Cold-junction temperature fluctuation	Directly affects $T_{h}$ through $T_{h}=T_{c}+\frac{E}{S}$	Keep cold junction under stable ambient conditions; introduce cold-junction compensation in future work
Voltage measurement uncertainty	Converted to temperature uncertainty as $\frac{u_{E}}{S}$	For S = 40.45 μV/°C, 1 μV corresponds to approximately 0.025 °C
Calibration fitting uncertainty	Affects the sensitivity used for temperature conversion	Evaluated from repeated heating–cooling cycles; sensitivity SD = 0.14 μV/°C
Reference temperature uncertainty	Affects the calibration baseline and comparison with reference thermocouples	Use the same calibration setup and compare with commercial K-type thermocouples
Lead heat conduction	Disturbs the local temperature field near the sensing junction	Optimize electrode geometry and reduce unnecessary lead thermal paths
Spatial temperature gradient	Causes deviation between the reference point and sensing junction	Place the reference thermocouple adjacent to the thin-film thermocouple

## Data Availability

The data presented in this study are available on request from the corresponding author.
